# Supplementary material for: Quantifying the kinetics of hematocrit and platelet count during febrile phase to develop a scoring system for predicting dengue shock syndrome in adults: A matched case - control study from a Hospital in Viet Nam
Source: PLoS Negl Trop Dis. 2026 Apr 20;20(4):e0014245. doi: 10.1371/journal.pntd.0014245 (PMC13108877; doi:10.1371/journal.pntd.0014245)
Supplement: S2 Table — (DOCX) [file pntd.0014245.s007.docx]

S2 Table. Factors associated with the development of DSS.

|  | Unadjusted effects | | Adjusted effects | |
| --- | --- | --- | --- | --- |
|  | OR  (95%CI) | p^(*)^ | OR  (95%CI) | p^(*)^ |
| Previous dengue | 1.59  (0.70 – 3.56) | 0.262 |  |  |
| Obesity | 1.67  (1.07 – 2.59) | 0.024 | 1.14  (0.77 – 1.69) | 0.505 |
| Number of clinical warning signs | 3.64  (2.65 – 4.99) | <0.001 | 3.04  (2.03 – 4.56) | <0.001 |
| HIR | 1.18  (1.13 – 1.23) | <0.001 | 1.14  (1.07 – 1.22) | <0.001 |
| PDR | 1.06  (1.04 – 1.07) | <0.001 | 1.04  (1.02 – 1.06) | <0.001 |
