# Supplementary material for: Quantifying the kinetics of hematocrit and platelet count during febrile phase to develop a scoring system for predicting dengue shock syndrome in adults: A matched case - control study from a Hospital in Viet Nam
Source: PLoS Negl Trop Dis. 2026 Apr 20;20(4):e0014245. doi: 10.1371/journal.pntd.0014245 (PMC13108877; doi:10.1371/journal.pntd.0014245)
Supplement: S3 Table — (DOCX) [file pntd.0014245.s008.docx]

S3 Table. Point-based score of three independent predictors of DSS identified by multivariate regression.

|  | Coefficient (*) | P | OR | 95%CI | DSS Score point |
| --- | --- | --- | --- | --- | --- |
| HIR ≥ 5% | 1.59 | < 0.001 | 4.91 | 2.21 – 10.88 | 3 |
| PDR ≥ 50% | 1.91 | < 0.001 | 6.74 | 3.06 – 14.88 | 4 |
| Number of clinical WS ≥ 2 | 3.06 | < 0.001 | 21.28 | 8.82 – 51.35 | 6 |

(*) Using conditional stepwise regression

OR: odd ratio, DSS: dengue shock syndrome, HIR: Hematocrit increase rate, PDR: Platelet decrease rate, WS: warning sign.
