# Supplementary material for: Quantifying the kinetics of hematocrit and platelet count during febrile phase to develop a scoring system for predicting dengue shock syndrome in adults: A matched case - control study from a Hospital in Viet Nam
Source: PLoS Negl Trop Dis. 2026 Apr 20;20(4):e0014245. doi: 10.1371/journal.pntd.0014245 (PMC13108877; doi:10.1371/journal.pntd.0014245)
Supplement: Attachment — Submitted filename: PNTD-D-25-01958_Reviewer_report.pdf [file pntd.0014245.s010.pdf]

REVIEWER REPORT

Journal: PLOS Neglected Tropical Diseases
Manuscript ID: PNTD-D-25-01958
Title: *Quantifying the Kinetics of Hematocrit and Platelet Count During Febrile Phase to Develop a Scoring System for Predicting Dengue Shock Syndrome in Adults*
Recommendation: Minor Revision
Date: 7 December 2025

SUMMARY FOR EDITORS

This is a well-written and clinically meaningful study addressing an important gap in dengue management: the lack of quantitative criteria for the WHO 2009 laboratory warning sign and the need for practical tools to predict dengue shock syndrome (DSS). Using a matched-case design (112 DSS, 336 non-DSS), the authors define useful thresholds for hematocrit increase (≥5%) and platelet decrease (≥50%) and develop a bedside scoring system with excellent discrimination (AUC 0.93).

The study is methodologically sound, with clear clinical implications. However, several methodological clarifications and an expanded discussion of limitations—especially the lack of model validation—are required before publication.

GENERAL ASSESSMENT

Strengths

- Addresses a high-priority clinical and WHO-identified research gap
- Strong matched-case observational design
- Large sample size for adult DSS (n=112)
- Clear dynamic characterization of hematocrit and platelet kinetics
- Uses routine laboratory parameters, enhancing global applicability
- Score is simple, intuitive, and has excellent predictive performance
- Presentation is generally clear with good figures and supplementary materials

Main Weaknesses

- No internal or external validation of the scoring system
- Single-center dataset with very specific population characteristics
- Some methodological steps (especially score derivation and matching) need clearer justification
- Limited discussion of obesity finding despite statistical significance
- Minor inconsistencies, formatting issues, and unclear footnotes

MAJOR COMMENTS

1. Lack of internal and external validation (critical)

The DSS score is not validated in an independent dataset. This must be clearly acknowledged.

Required actions:

- Expand discussion on this limitation (risk of overfitting, reduced generalizability).
- Clarify why validation was not feasible (e.g., sample size constraints, single-epidemic dataset).
- Explicitly state that **external validation is needed before widespread implementation**.
- Soften recommendations for adoption accordingly (e.g., "may assist" rather than "should guide").

---

2. Scoring system derivation requires justification

The manuscript states that regression coefficients were multiplied by 2 and rounded to create integer point values. This approach appears arbitrary.

Required actions:

- Provide rationale for this specific transformation (e.g., clinical simplicity, alignment with prior clinical scores).
- Cite methodological precedents such as Sullivan et al., *Stat Med.* 2004;23:1631–1660.
- State whether other approaches (e.g., dividing by smallest coefficient) were considered.

---

3. Matching and statistical methods need clearer explanation

The design is appropriate, but several analytic choices require more transparency.

Clarify:

- Whether matching was performed prospectively or retrospectively.
- Why the 1:3 ratio was used (availability vs. power considerations).
- Handling of missing data (if any) for HIR, PDR, or WSs.
- Assumptions behind using % change rather than absolute change for HIR/PDR.
- Rationale for using the Youden index for cut-off selection.
- Whether collinearity and model calibration were assessed.

Providing this information will substantially strengthen methodological credibility.

4. Obesity finding is statistically significant but under-discussed

Obesity (38% DSS vs 27% non-DSS) is significant ($p = 0.03$), yet receives minimal discussion.

Expand discussion:

- Place finding in context of existing dengue–obesity literature.
- Discuss plausible mechanisms (endothelial dysfunction, metabolic inflammation).
- State whether obesity remained significant in multivariate modeling.
- If excluded from final model, explain why (collinearity? weak adjusted effect?).

---

5. Interpretation of risk groups and monitoring recommendations

The manuscript suggests specific monitoring frequencies per risk group.

Clarify whether these are:

- Derived from WHO guidelines,
- Local protocols,
- Expert opinion, or
- Empirically supported by your dataset.

If based on expert opinion, soften tone and state this clearly.

---

6. Limitations section needs expansion

The limitations around model validation, single-center dataset, and possible selection bias should be strengthened.

Please add discussion of:

- No internal/external validation
- Single-center design
- Differences between retrospective and prospective cohorts
- Potential inter-observer variability in WS assessments
- Overfitting risk in a 3-parameter model
- Hydration status and laboratory variability influencing HIR/PDR
- Selection bias introduced by matching based on CBC day

This will bring the manuscript closer to PLOS reporting expectations.

MODERATE COMMENTS

7. Comparison with Lam et al.

The discussion comparing your results to Lam et al. (2017) can be enhanced.

Strengthen by noting:

- Adult vs pediatric physiology (hematocrit dynamics differ).
- Lam's inclusion of severe bleeding cases, which depress hematocrit.
- Impact of using **absolute values** in Lam vs **dynamic changes (HIR)** in your study.

---

8. Baseline characteristics table improvements

Table 1 needs refinements for clarity.

Required corrections:

- Add p-value for pregnancy or explain its absence.
- Include units for AST/ALT.
- Add "IQR: interquartile range" in footnotes.
- Define abbreviations consistently (e.g., WS, BMI).

---

9. Calculator accessibility

The online calculator is helpful but needs permanence.

Recommendations:

- Confirm that the link is accessible outside your institution.
- Consider archiving code or a static version (GitHub, Zenodo, Figshare).
- Provide screenshots or a supplementary example.

MINOR COMMENTS

Abstract

- Include 95% CI for AUC, sensitivity, specificity.
- Consider simplifying phrasing ("concurrent rapid decrease").

Language and Style

- Correct "subsenquently" → **subsequently**.
- Remove duplicate words ("become become overwhelmed").
- Standardize superscripts ($\times 10^9$/L), percent formatting, and spacing.

Methods

- Clarify timing of "daily CBC"—same time each day?
- Provide manufacturer locations for diagnostic kits (NS1 rapid test, IgM ELISA).
- Note that defining adults as ≥16 differs from WHO norms.

Results

- Emphasize physiologic basis of HIR/PDR accelerating on days 3–5.
- Correct minor typographical inconsistencies.

Figures

- Increase font sizes in Figures 2 and 3.
- Add a supplementary schematic showing score calculation with a sample patient.

Tables

- For Table 2: consider adding a "Clinical Action" column.

Supporting Information

- Ensure all S tables and figures are cited in main text.

References

- Ensure all access dates are provided (e.g., WHO fact sheets).
- Verify reference formatting for recency and consistency.

QUESTIONS FOR AUTHORS

1. Were systematic differences observed between prospective and retrospective datasets?
2. Was inter-rater reliability assessed for warning signs?
3. Did you consider including other routine labs (WBC, albumin)?
4. Did obesity remain significant after multivariate analysis?
5. Were CBC measurements consistently timed each day?
6. Has the score been piloted prospectively?
7. Were there seasonal severity patterns during the study period?

ETHICAL AND DATA ACCESS NOTES

- Confirm that the ethics committee approved the waiver of consent for retrospective data.
- Consider sharing de-identified data in a public repository to allow others to validate your score.

CONCLUSION

This is a strong and clinically impactful study that addresses an important gap in dengue management. The methodology is appropriate, findings are logically presented, and the proposed score is promising for real-world triage. The primary limitations—lack of validation and single-center design—require clearer framing, but do not negate the value of the study.

Recommendation: Minor Revision

After addressing the clarifications and strengthening the methodological rationale, this manuscript will make a valuable contribution to PLOS Neglected Tropical Diseases.
