# Supplementary material for: Quantifying the kinetics of hematocrit and platelet count during febrile phase to develop a scoring system for predicting dengue shock syndrome in adults: A matched case - control study from a Hospital in Viet Nam
Source: PLoS Negl Trop Dis. 2026 Apr 20;20(4):e0014245. doi: 10.1371/journal.pntd.0014245 (PMC13108877; doi:10.1371/journal.pntd.0014245)
Supplement: Attachment — Submitted filename: REVIEW1.docx [file pntd.0014245.s009.docx]

Dengue fever, a global public health challenge, infects approximately 100 to 400 million people annually. The massive number of cases often overwhelms local health systems during epidemic seasons, making early prediction of dengue shock syndrome (DSS) a critical issue. However, the lack of consensus on the definition of the laboratory warning sign "elevated hematocrit with rapid thrombocytopenia" complicates the use of all warning signs for DSS prediction. In this study, Vu Thi Thanh Ma et al developed a risk score system for predicting the risk of adult dengue shock syndrome (DSS) by quantifying the changes of hematocrit and platelet count during the fever stage of dengue fever patients, which can provide a scientific basis for clinical triage and management.
The primary challenge lies in accurately quantifying changes in hematocrit and platelet count, and establishing their correlation with DSS onset. Furthermore, given the time-sensitive nature of DSS development, capturing these changes during early disease stages and transforming them into actionable predictive tools remains a significant hurdle. Another key challenge involves designing matched case-control studies to ensure data reliability and result generalizability while minimizing confounding factors' impact on analysis.

The study has several limitations. Firstly, data on dengue virus serotypes, viral load, and IgG/IgM status were lacking, which may influence the risk of shock and the author should provide the above information

. Secondly, although the cohort of 112 DSS and 336 non-DSS patients provided sufficient power for analysis, the sample size was not pre-calculated . Thirdly, requiring at least two consecutive complete blood count (CBC) results increased workload and treatment costs, which may limit the applicability of the DSS score in some settings . Fourthly, the study was single-centered, and the scoring system has not yet been validated .

It is necessary to describe the course days of the two groups ( DSS group and non-DSS group ) in results.The picture content of Fig.2 and Fig.3 is inconsistent with the description of the caption. The small title in the figure shows the parameter values of a day after the onset, and the content expressed is the continuous change of parameter values of different days.

We cannot know when the two parameters are used for differential diagnosis of DSS.The description of the results did not explain when the parameters were used to diagnose DSS after the onset of the patient, and how much time to diagnose DSS earlier than other methods.

According to the World Health Organization's 2009 guidelines, dengue fever is classified into three categories: dengue fever without warning signs, dengue fever with warning signs, and severe dengue fever. Dengue shock syndrome (DSS) accounts for approximately 80% of severe dengue fever cases. Although existing studies have explored the relationship between warning signs and DSS, there remains controversy regarding the precise definition of the specific sign "elevated hematocrit with rapid decline in platelet count." Additionally, previous research has predominantly focused on pediatric patients, with limited studies on adults, and there is a lack of systematic scoring tools to integrate multiple warning signs for risk assessment.
 Case-control study design. The study was conducted at Ho Chi Minh City Tropical Disease Hospital in Vietnam, with samples drawn from adult dengue fever patients hospitalized between November 2022 and August 2023. Each dengue shock syndrome (DSS) patient was matched with three non-DSS patients based on age (±5 years), gender, same-day complete blood count (CBC), and pregnancy status. Priority was given to selecting control group members whose admission dates, disease duration, and ward assignments were closest to those of the DSS patients.
Data Collection and Analysis: Data analysis was performed using IBM SPSS Statistics 27. Conditional logistic regression was applied to compare data between the DSS and non-DSS groups, with p<0.05 indicating statistical significance. The regression model employed a conditional stepwise estimation method, and diagnostic performance was evaluated through sensitivity, specificity, and likelihood ratio. The final regression model was converted into a practical scoring system, where coefficients of adjusted variables were multiplied by 2 and rounded to obtain corresponding point values.
A total of 448 dengue fever patients were enrolled, including 112 with DSS and 336 matched non-DSS controls. The median age was 25 years, with a male-to-female ratio of 1.3:1. Most patients (80%) were hospitalized on days 3 and 4 of the illness. Common symptoms included fever (100%), myalgia (72%), persistent vomiting (58%), and abdominal pain or tenderness (50%). Obesity was more prevalent in DSS cases (38% vs. 27%, p = 0.03).
In non-DSS patients, hematocrit (HbO) showed a slight increase from day 2, peaked on day 5, and then declined. In contrast, DSS patients experienced a rapid rise in hematocrit 1-2 days before shock onset. Both groups began showing a decline in platelet count from day 2, with DSS patients demonstrating more pronounced decreases between days 3 and 5. Notably, DSS patients exhibited significantly higher hematocrit increase rate (HIR) and platelet reduction rate (PDR) from day 3 to day 5 compared to non-DSS patients.
Using the Youden index, we selected HIR ≥ 5% and PDR ≥ 50% as cutoff thresholds. Under these conditions, the AUC values were 0.79 (95% CI: 0.74–0.84) and 0.81 (95% CI: 0.78–0.86), respectively. The combined AUC for HIR ≥ 5% and PDR ≥ 50% was 0.81 (95% CI: 0.77–0.85), with a sensitivity of 69.7% and specificity of 83.0%.
Multivariate regression analysis of the prediction scoring system identified three independent DSS predictors (p <0.001): HIR ≥ 5% (coefficient 1.59), PDR ≥ 50% (1.91), and ≥ 2 clinical warning signs (3.06). These factors were converted into a point-based scoring system: HIR ≥ 5% scored 3 points, PDR ≥ 50% scored 4 points, and ≥ 2 WS scored 6 points. The scoring system classified risks into low risk (≤ 4 points), moderate risk (6-7 points), and high risk (≥ 9 points).
Comparative analysis with other studies: The median age of adult patients in this study was 25 years, younger than those in Singapore or Taiwan studies. Most DSS events (95%) occurred between days 4 to 6 of the illness, peaking on day 5. This underscores the importance of closely monitoring clinical and laboratory parameters starting from days 2-3. Hematocrit levels rose significantly faster in DSS patients than in non-DSS patients 1-2 days before shock onset. Thrombocytopenia, a recognized feature of dengue fever, may result from reduced bone marrow production and increased peripheral platelet destruction.
Multivariate analysis identified three independent DSS predictors: ≥ 2 clinical warning signs, HIR ≥ 5%, and PDR ≥ 50%. The simplified logistic regression model incorporating these factors demonstrated excellent predictive performance (AUC = 0.93,95% CI: 0.90–0.96). The scoring system is user-friendly and can be quickly implemented for bedside risk assessment via mental calculation or a small spreadsheet on smart devices.
 It is recommended to specify the WHO 2009 laboratory warning signal as "a hematocrit increase of ≥5% and a platelet count decrease of ≥50% compared to the previous day." The DSS risk score can classify patients into low, medium, and high-risk groups for appropriate monitoring and management. Future validation of this scoring system is needed in other locations, outpatient settings, and across different age groups (including children under 16 and adults over 60) to achieve broader clinical application.
This study employs a dynamic approach to compare blood cell counts and platelet counts over two consecutive days, providing precise characterization of individual variations. The DSS scoring system is user-friendly and facilitates the reallocation of clinical resources, reducing unnecessary workload for low-risk patients. This method proves more practical than the current recommendation of monitoring all patients with warning signs every 2-4 hours.

-
