# Supplementary material for: Quantifying the kinetics of hematocrit and platelet count during febrile phase to develop a scoring system for predicting dengue shock syndrome in adults: A matched case - control study from a Hospital in Viet Nam
Source: PLoS Negl Trop Dis. 2026 Apr 20;20(4):e0014245. doi: 10.1371/journal.pntd.0014245 (PMC13108877; doi:10.1371/journal.pntd.0014245)
Supplement: Attachment — Submitted filename: Response to Reviewers.docx [file pntd.0014245.s012.docx]

**Dear Editor and Reviewers,**

We sincerely thank you for your valuable comments and suggestions, which have greatly helped us improve our manuscript. Below, we provide our detailed responses to each comment from the Editor and reviewers.

**REVIEWER #1**

***Comment 1:*** The line 152 should be: “in the febrile ( PltDn−1− Plt Dn) phase. PDR (%) = (PltDn−1− Plt Dn) / (PltDn−1) x100%.

*Response:* Thank you for this helpful suggestion. We have revised line 152 accordingly. The manuscript now reads: “Hematocrit increase rate (HIR) is defined as the percentage of Hct increase between two consecutive days in the febrile phase of dengue” *(page 8, line 153 - 154)* and “Platelet decrease rate (PDR) is defined as the percentage of Plt decrease between two consecutive days in the febrile phase of dengue.” *(page 8, line 155 - 156).*

**REVIEWER #2**

*We sincerely thank Reviewer #2 for your thoughtful and constructive comments. We have carefully considered each suggestion and revised our manuscript accordingly. Below, we provide a detailed point‑by‑point response to all comments.*

***Comment 1:*** In the methodology section, it is necessary to describe in detail why the case-control ratio was chosen.

*Response:* We selected a 1:3 case-to-control ratio based on prior evidence indicating that while statistical power increases with more controls, precision gains plateau beyond this ratio. Thus, 1:3 was considered the optimal balance between feasibility and efficiency (Katki et al., BMC Med Res Methodol. 2023;23:153). The explanation has been added in the manuscript *(page 9, line 168 - 169).*

***Comment 2:*** It is necessary to describe the course days of the two groups (DSS group and non-DSS group) in results.

*Response:* We appreciate this suggestion. We have added details in the manuscript. Specifically, the most common admission day was day 4 of illness (43.97%), the defervescence days showed no difference between DSS and non-DSS groups, and shock occurred mainly on day 5 (55.36%) *(page 11, table 1).*

***Comment 3:*** The picture content of Fig.2 and Fig.3 is inconsistent with the description of the caption.

*Response:* We have revised the captions to ensure accuracy. In the manuscript, Fig. 2 is now titled “Kinetics of hematocrit (above) and HIR (below) in DSS and non-DSS cases during consecutive days of illness before shock onset”, and Fig. 3 is titled “Kinetics of platelet count (above) and HDR (below) in DSS and non-DSS cases during consecutive days of illness before shock onset”.

***Comment 4:*** The description of the results did not explain when the parameters were used to diagnose DSS after the onset of the patient, and how much time to diagnose DSS earlier than other methods.

*Response:* We used the two parameters to calculate the risk of DSS occurrence, not for the differential diagnosis of DSS. Therefore, we did not compare our approach with other methods addressingthat subject. Depending on wheather the risk of DSS was low, intermediate, or high, we developed a framework for the clinical monitoring of dengue patients.

**REVIEWER #3**

*We would like to express our sincere gratitude to Reviewer #3 for the insightful and constructive comments. We have thoroughly considered each point raised and incorporated the necessary revisions to strengthen the manuscript. A detailed, point‑by‑point response is provided below.*

***Comment 1:*** The DSS score is not validated in an independent dataset. This must be clearly acknowledged.

*Response:* In response to Reviewer 3’s comment regarding validation, we would like to express our sincere appreciation for highlighting this important aspect. We have subsequently undertaken internal validation using cross‑validation and bootstrap techniques within SPSS. Our logistic regression model (including HIR, PDR, and warning signs) was robustly validated using 5‑fold cross‑validation (accuracy 87.4%, specificity 93.0%, AUC 0.93) and bootstrap resampling, while the DSS score was assessed through calibration analysis, showing good agreement between predicted and observed outcomes. In the manuscript, the Methods section now describes the internal validation approach *(page 9, line 174 - 176*), the Results section reports the validation outcomes *(page 13, line 232 – 237; page 14, line 252 - 255*), and the Discussion section acknowledges the limitation of not yet performing external validation *(page 18, line 351 - 356*). Accordingly, we have softened our recommendations for clinical adoption, indicating that the score may assist clinicians in risk stratification and bedside decision-making, rather than suggesting it should guide practice.

***Comment 2:*** The manuscript states that regression coefficients were multiplied by 2 and rounded to create integer point values. This approach appears arbitrary.

*Response:* In developing the score, we doubled and rounded the regression coefficients to obtain integer values, aiming to enhance clinical simplicity and bedside usability. This approach is consistent with methods used in other risk scores (e.g., Framingham). We have added this explanation and reported the transformation in the Results section of the manuscript *(page 13, line 241 - 243*).

***Comment 3:*** Whether matching was performed prospectively or retrospectively.

*Response:* We appreciate the opportunity to clarify this issue. In the manuscript, we have specified that matching was performed in both retrospective and prospective phases. In the prospective phase, eligible patients were enrolled and matching was conducted after data collection, based on predefined criteria. In the retrospective phase, dengue shock cases were identified first and then matched with non-shock controls concurrently with data collection. We also clarified that prospective matching stopped once three controls were assigned per case, prioritizing similarity in admission time and hospital ward. These revisions ensure that the methodology is transparent and reproducible *(page 7, line 122 - 127*).

***Comment 4:*** Why the 1:3 ratio was used (availability vs. power considerations).

*Response:* We selected a 1:3 case-to-control ratio based on prior evidence indicating that while statistical power increases with more controls, precision gains plateau beyond this ratio. Thus, 1:3 was considered the optimal balance between feasibility and efficiency (Katki et al., BMC Med Res Methodol. 2023;23:153). The explanation has been added in the manuscript *(page 9, line 168 - 169).*

***Comment 5:*** Handling of missing data (if any) for HIR, PDR, or WSs.

*Response:* We acknowledge the comment, but there were no missing values in HIR, PDR, and WSs because cases without complete data for these variables were not eligible for inclusion according to our selection criteria. All enrolled patients had daily complete blood count (CBC) monitoring, which ensured data completeness for these parameters.

***Comment 6:*** Assumptions behind using % change rather than absolute change for HIR/PDR.

*Response:* Because baseline hematocrit and platelet values vary widely among patients and are often unknown at the time of hospital admission, absolute changes may not reflect clinically relevant shifts. We therefore used percentage change for each patient to provide a standardized measure of relative variation, which we believe is more suitable for defining HIR and PDR in our dataset. This rationale was considered during analysis but not added to the manuscript.

***Comment 7:*** Rationale for using the Youden index for cut-off selection.

*Response:* The Youden index was selected because it provides a balanced criterion that maximizes the sum of sensitivity and specificity. This method is widely used in diagnostic research and was deemed appropriate for identifying clinically meaningful thresholds in our study.

***Comment 8:*** Whether collinearity and model calibration were assessed.
Providing this information will substantially strengthen methodological credibility.

*Response:* We have provided additional details in the manuscript and added information on model calibration in the Supporting infromation to strengthen methodological credibility (*page 14, line 252 - 255, S3 Fig and S4 Fig).*

***Comment 9:*** Obesity (38% DSS vs 27% non-DSS) is significant (p = 0.03), yet receives minimal discussion.

*Response:* In response to this comment, obesity showed a significant difference in univariate analysis but was not significant in multivariate analysis. As this was not a primary objective and the study was not designed to assess the association between obesity and DSS, we did not discuss it further. We have added the multivariate results in the revised Results section *(page 10, line 190 - 192).*

***Comment 10:*** Interpretation of risk groups and monitoring recommendations

*Response:* To clarify this point, we have now included that the proposed monitoring frequencies are empirically supported by our dataset. Based on the diagnostic performance of the DSS score, we derived a practical framework to triage patients into low, intermediate, and high‑risk groups, with corresponding monitoring intervals ranging from outpatient follow‑up for very low‑risk patients to intensive bedside monitoring for high‑risk patients. These suggestions are based on our own clinical experience. They are preliminary and will require validation in larger prospective studies *(page 18, line 340 - 344)****.***

***Comment 11:*** The limitations around model validation, single-center dataset, and possible selection bias should be strengthened.

*Response:* This point has been addressed in the revised version. We have expanded the Limitations section to acknowledge that, although internal validation including bootstrapping demonstrated good predictive ability of the DSS score, external validation is still lacking. We also note the theoretical risk of overfitting in a three‑parameter model, the single‑center design limiting generalizability, potential inter‑observer variability in retrospective warning sign assessments, the influence of hydration status and laboratory variability on HIR/PDR and selection bias introduced by matching based on CBC day. These additions strengthen transparency and highlight the need for further multicenter, prospective validation *(page 19, line 370 - 375).*

***Comment 12:*** The discussion comparing your results to Lam et al. (2017) can be enhanced.

*Response:* In the Discussion, we have already noted differences related to absolute values versus dynamic changes (HIR) and study design. We will further strengthen this section by adding comments on physiological differences between adults and children, as well as Lam’s inclusion of severe bleeding cases that may depress hematocrit *(page 15, line 282 - 285).*

***Comment 13:*** Baseline characteristics table improvements. Table 1 needs refinements for clarity.

*Response*: Pregnancy involved only one DSS case matched with three controls; therefore, calculating a p‑value is not meaningful. Units for AST/ALT (U/L) are already indicated in the table. We will add the definition of IQR (interquartile range) in the footnotes and ensure consistent abbreviation definitions as requested *(page 11, table 1)*

***Comment 14:*** The online calculator is helpful but needs permanence.

*Response*: We confirm that the online calculator link is accessible outside our institution. To ensure permanence, we have archived the code and provide a static version on an open Zenodo repository, available at <https://doi.org/10.5281/zenodo.18513829> or <https://sites.google.com/pnt.edu.vn/infectiousdiseases-dss-score>. In addition, we have added screenshots and a supporting example in the manuscript to facilitate use *(page 19, line 382, S5 Fig).*

***Comment 15:*** Requests comprehensive refinements to improve clarity and consistency, including adding statistical details, correcting language and formatting, clarifying methods, emphasizing physiologic rationale in results, improving figures and tables, ensuring proper citation of supplementary materials, and standardizing references.

*Response*:

We retained the phrase “concurrent rapid decrease” to reflect the WHO wording of warning signs. The NS1Ag and MAC ELISA Dengue kits used were those available at the Hospital for Tropical Diseases, where the study was conducted. Regarding Table 2, we did not add a “Clinical Action” column since such actions are only preliminary suggestions derived from our data, whereas Table 2 presents objective study findings. All other suggested corrections will be incorporated as requested.

- **Abstract:** Added 95% CI for AUC, sensitivity, and specificity.
- **Language and Style:** Corrected typographical errors, removed duplicate words, and standardized formatting (superscripts, percentages, spacing).
- **Methods:** Clarified timing of daily CBC and noted that defining adults as ≥16 differs from WHO norms.
- **Results:** Emphasized the physiologic basis of HIR/PDR acceleration on days 3–5 and corrected minor inconsistencies.
- **Figures:** Increased font sizes in Figures 2 and 3 and added a supplementary schematic illustrating score calculation with a sample patient.
- **Supporting Information:** Ensured all supplementary tables and figures are cited in the main text.
- **References:** Updated access dates and standardized formatting for consistency and recency.

These revisions improve clarity, methodological transparency, and overall readability of the manuscript.

**Reviewer 3’s questions and our responses:** Were systematic differences observed between prospective and retrospective datasets?

*Response*: No major systematic differences were observed; CBC results and vital signs are objective laboratory measurements, thus not influenced by whether data were collected prospectively or retrospectively.

2. Was inter-rater reliability assessed for warning signs?

*Response*: We acknowledge this limitation. In the retrospective dataset, warning signs relied on clinicians’ judgment, while in the prospective dataset they were jointly assessed with the study team for greater accuracy; Further prospective validation of the scoring system is needed.

3. Did you consider including other routine labs (WBC, albumin)?

*Response*: Routine CBC data, including WBC, were available. Leukopenia is common in dengue but not directly associated with DSS, and our analysis showed no difference in WBC decline between shock and non‑shock groups. Serum albumin was only measured in a few recurrent shock cases, so data were insufficient for analysis.

4. Did obesity remain significant after multivariate analysis?

*Response*: Obesity did not remain significant after multivariate analysis, with no difference observed between the two patient groups.

5. Were CBC measurements consistently timed each day?

*Response*: As this was an observational study, CBCs were performed according to clinicians’ orders, usually at admission and each morning thereafter.

6. Has the score been piloted prospectively?

*Response:* The score has not yet been piloted prospectively.

7. Were there seasonal severity patterns during the study period?

*Response:* We did not observe seasonal severity patterns during the study period.

**REVIEWER #4**

*We sincerely thank Reviewer #4 for the thoughtful questions and constructive feedback, which have helped us improve the manuscript. We have carefully addressed each question raised by Reviewer #4.* *Below are our point‑by‑point responses to the Reviewer #4*

***Comment 1:*** How do you analyze the blood examinations for a patient who had more than one result in a single day?

*Response*: For patients with more than one CBC result in a single day, we analyzed the earliest value obtained that day to ensure randomization and avoid any intentional selection bias. This has been revised accordingly *(page 7, line 134 - 135).*

***Comment 2:*** What about data you collected retrospectively? How did you record the diagnosis of DSS? Was it based on the medical record without any further analysis? For example, after analyzing the case carefully, you found that the patient actually did not experience shock.

*Response*: To clarify this point, the data we collected were based on what the treating clinicians wrote in the medical records. The treating doctors always described the clinical signs and hematocrit when the DSS diagnosis was made. We did not observe any cases of mistaken DSS diagnosis in our study population.

***Comment 3:*** According to your research, when is the scoring system applicable? Is the scoring system applicable on the second, third, or fourth day? Do you believe that sensitivity and specificity could vary? Please discuss this.

*Response*: In response to this comment, our study showed that DSS typically occurred between day 4 and day 7 of illness (with day 1 defined as the first day of fever). Therefore, we recommend applying the DSS score as early as day 2 or day 3 and repeating the assessment daily from the time of dengue diagnosis to predict risk. Because only 14 of 112 cases developed shock on day 4, compared with 62 of 112 on day 5, it was not feasible to compare sensitivity and specificity across illness days.

**ADDITIONAL EDITOR COMMENTS**

*We sincerely thank the Editor for the additional comments and valuable suggestions, which have helped us further improve the manuscript.* *We have carefully addressed each of the Editor’s additional comments, and our point‑by‑point responses are presented below.*

***Comment 1:*** Matched case-control labeled as “matched-case observational study” is unclear—standard terminology is matched case-control. The tertiary referral hospital setting may introduce selection bias toward severe cases, so please check.

*Response*: We have changed the terminology to the standard usage, “matched case-control study,” in the manuscript *(page 2, line 26; page 6, line 103)* and article title has been updated to *“Quantifying the Kinetics of Hematocrit and Platelet Count During Febrile Phase to Develop a Scoring System for Predicting Dengue Shock Syndrome in Adults: A Matched Case - Control Study from a Hospital in Viet Nam”*

***Comment 2:*** Exclusions for prior anti-shock treatment, underlying thrombocytopenia/anemia, transfusions, and insufficient CBCs are logical to isolate natural progression; however, they exclude many severe cases, potentially underestimating risk factors.

*Response*: In the revised Discussion, we clarified our exclusion criteria and explained that patients were followed from admission without severe dengue until they either developed DSS or recovered, minimizing selection bias. We acknowledged that this may have excluded the most severe cases but allowed focus on natural progression of the disease. We emphasized that plasma leakage and shock predominate in adults, which was the primary focus, and noted that future studies should assess predictors of other severe forms such as hemorrhage, marked liver enzyme elevations, and organ dysfunction *(page 20, line 389 - 393).*

***Comment 3:*** Matching on age (±5 years), sex, same-day CBCs, and pregnancy is strong for confounding control. Additional prioritization by admission time, ward, and day is good. The 1:3 ratio is efficient, but multiple candidates introduce selection subjectivity.

*Response*: We acknowledge that when multiple candidates were available for the control group, subjectivity could be introduced. To minimize this, matching was performed randomly based on predefined priority criteria such as admission time and clinical ward, and stopped once three suitable controls were identified per case, without further selection. We have also added details of the matching process in the manuscript under *the Matching criteria* to ensure transparency *(page 7, line 122 - 127).*

***Comment 4:*** Adherence to 2019 Vietnam and 2009 WHO guidelines is consistent, though the 2009 WHO guideline is outdated. If possible, please update it.

*Response*: At the time of this study the 2019 Vietnam and 2009 WHO guidelines were the latest. We updated to the 2025 WHO guidelines in our discussion.

***Comment 5:*** Please add details about the ethical statement process for participants aged 16–17 years.

*Response*: We have revised the manuscript accordingly. Specifically, all patients in the prospective period provided written informed consent. For those aged 16–17 years, study information was explained to both patients and their parents/guardians; patients gave assent and parents/guardians provided written consent. In the retrospective period, data were extracted from medical records, and patient consent was waived by the Hospital’s Ethics Committee for Biomedical Research *(page 7-8, line 141 - 145).*

***Comment 6:*** Day 1 as fever onset is standard; defining ≥16 years as adult per policy is pragmatic. The DSS definition aligns with guidelines (narrow pulse pressure, cold extremities, etc.), but clinical diagnosis without echo confirmation of leakage may miss compensated shock.

*Response*: We completely agree that echography was not performed routinely and thus early plasma leakage might not always be detected. However, according to the Handbook for Clinical Management of Dengue (WHO, 2012 update), compensated shock can be reliably identified based on clinical signs such as rapid, weak pulse, cold extremities, and capillary refill time > 2 seconds. Therefore, the lack of daily ultrasound did not affect our ability to detect compensated shock, and we have not revised the Discussion.

***Comment 7:*** Conditional logistic regression is correct for the matched design; however:

• Stepwise selection risks overfitting/instability in small samples.

• Use of likelihood ratios over predictive values is wise given artificial 25% prevalence.

• Score development by doubling/rounding coefficients is common but arbitrary. Validation (internal/external) is missing.

• No sample size/power calculation reported.

• No adjustment for multiple comparisons or handling of missing data described.

*Response*: These points have been addressed in the revised version. We recognize that stepwise selection may risk overfitting in small samples; therefore, we have recently added validation using cross‑validation and bootstraps *(page 13, line 232 - 237)****.*** We clarified the rationale for rounding to integer scores *(page 13, line 241 - 243)* and noted that no missing data were present. Regarding sample size, our novel approach using the kinetics of hematocrit and platelet count lacked prior studies employing this approach. Therefore, as a pilot study, we empirically selected approximately 100 DSS and 300 non‑DSS cases.

Once again, we sincerely thank the reviewers and the Editor for your constructive comments and valuable suggestions, which have significantly improved our manuscript.

Sincerely,

Nghia Ho Dang Trung

On behalf of all authors

Department of Infectious Diseases, Pham Ngoc Thach University of Medicine

Ho Chi Minh City, Vietnam

Date: February 6th, 2026
