# Supplementary material for: Quantifying the kinetics of hematocrit and platelet count during febrile phase to develop a scoring system for predicting dengue shock syndrome in adults: A matched case - control study from a Hospital in Viet Nam
Source: PLoS Negl Trop Dis. 2026 Apr 20;20(4):e0014245. doi: 10.1371/journal.pntd.0014245 (PMC13108877; doi:10.1371/journal.pntd.0014245)
Supplement: Attachment — Submitted filename: Response to Reviewers (2).docx [file pntd.0014245.s013.docx]

**Dear Editor and Reviewers,**

We sincerely thank you for the careful evaluation of our revised manuscript. We have addressed all comments from the first round of review, and we are grateful that the reviewers recognize the clarity of our objectives, the appropriateness of the study design, and the transparency of our analyses and results.

**Response to Reviewer 2:**

We sincerely thank Reviewer 2 for the detailed and constructive evaluation of our revised manuscript. We appreciate the recognition of the study’s clearly defined objectives, the appropriateness of the matched case-control design, and the consistency between the analytical plan, methods, and results. We have clarified in the manuscript that the statistical analyses implicitly test the predictive value of HIR, PDR, and their combination with clinical WS, in line with the standards of observational studies.

We acknowledge the reviewer’s comments on the limitations related to the single-center, partially retrospective design and lack of external validation, and we have emphasized these points in the discussion. Future directions, including multicenter validation, inclusion of pediatric/adolescent populations, and assessment of seasonal or geographic variability, have been highlighted.

We are encouraged by the reviewer’s assessment that the study advances DSS prediction by transforming vague clinical criteria into quantifiable metrics, thereby enabling precision triage and improving patient outcomes. We believe the revised manuscript now fully addresses the reviewer’s concerns and reflects strong scientific merit, methodological rigor, and clinical applicability.

**Response to Reviewer 4:**

We sincerely thank Reviewer 4 for the valuable suggestion. In line with your recommendation, we have revised the manuscript to emphasize that the DSS score may be applied as early as day 2 or day 3. This point has been incorporated at the end of the discussion section *(page 18, line 343 - 344)* and reiterated in the conclusion *(page 20, line 399 - 400)*, highlighting its potential utility for early triage and timely clinical decision-making. We believe this addition strengthens the practical applicability of our findings and directly addresses your comment.

Sincerely,

Nghia Ho Dang Trung

On behalf of all authors

Department of Infectious Diseases, Pham Ngoc Thach University of Medicine

Ho Chi Minh City, Vietnam

Date: February 20th, 2026
